# Interactive imitation learning for dexterous robotic manipulation: challenges and perspectives—a survey

**DOI:** 10.3389/frobt.2025.1682437

**Published:** 2025-12-19

**Authors:** Edgar Welte, Rania Rayyes

**Affiliations:** AI and Robotics (AIR), Institute of Material Handling and Logistics (IFL), Karlsruhe Institute of Technology (KIT), Karlsruhe, Germany

**Keywords:** dexterous manipulation, review, imitation learning, interactive learning, human-in-the-loop learning, learning from demonstration

## Abstract

Dexterous manipulation is a crucial yet highly complex challenge in humanoid robotics, demanding precise, adaptable, and sample-efficient learning methods. As humanoid robots are usually designed to operate in human-centric environments and interact with everyday objects, mastering dexterous manipulation is critical for real-world deployment. Traditional approaches, such as reinforcement learning and imitation learning, have made significant strides, but they often struggle due to the unique challenges of real-world dexterous manipulation, including high-dimensional control, limited training data, and covariate shift. This survey provides a comprehensive overview of these challenges and reviews existing learning-based methods for real-world dexterous manipulation, spanning imitation learning, reinforcement learning, and hybrid approaches. A promising yet underexplored direction is interactive imitation learning, where human feedback actively refines a robot’s behavior during training. While interactive imitation learning has shown success in various robotic tasks, its application to dexterous manipulation remains limited. To address this gap, we examine current interactive imitation learning techniques applied to other robotic tasks and discuss how these methods can be adapted to enhance dexterous manipulation. By synthesizing state-of-the-art research, this paper highlights key challenges, identifies gaps in current methodologies, and outlines potential directions for leveraging interactive imitation learning to improve dexterous robotic skills.

## 1 Introduction

Recent advances in robot hardware and learning algorithms have led to a surge of interest in dexterous manipulation as a key area of robotics research. Whether in the context of humanoid robots interacting in human environments, or robotic hands performing precise object manipulations, dexterous manipulation presents unique challenges due to its high-dimensional action spaces, complex kinematics, and intricate contact dynamics (Zhu et al., 2019; Kadalagere Sampath et al., 2023; Yu and Wang, 2022). These factors make learning-based approaches notably appealing. As the dimensionality of the action space increases, the amount of training data required grows exponentially (Sutton and Barto, 2018; Lu et al., 2024; Kubus et al., 2018), making sample-efficient learning methods increasingly crucial. Imitation learning has emerged as an effective strategy for dexterous manipulation, enabling robots to learn complex skills by mimicking human demonstrations (Mikami, 2009). Leveraging recorded training data to learn a policy via imitation learning offers high sample efficiency, especially compared to reinforcement learning approaches where the policy is developed independently through interaction with the environment (Radosavovic et al., 2021; Hu et al., 2023). This efficiency is particularly valuable in real-world scenarios, where frequent and random interactions with the environment can be both hazardous and costly (Sutton and Barto, 2018; Wang et al., 2022; Han et al., 2023). However, supervised imitation learning like Behavioral Cloning (BC) is known to suffer from a covariate shift, leading to a mismatch between the state distribution in the training data and the distribution encountered during the execution of the trained policy (Sun et al., 2023). Interactive Imitation Learning (IIL) offers a promising solution to address this challenge by integrating real-time human feedback into the learning process, effectively combining imitation learning with interactive machine learning techniques (Celemin et al., 2022; Amershi et al., 2014).

**FIGURE 1:**
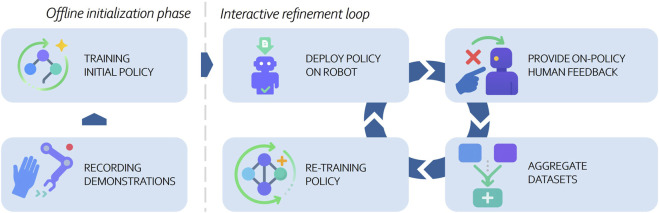
Overview of the interactive imitation learning (IIL) workflow. The process begins with an offline initialization phase consisting of demonstration collection and initial policy training. The resulting policy is then iteratively refined through an interactive loop in which the robot executes the policy, a human provides on-policy feedback, the new data are aggregated, and the policy is retrained.

Unlike standard imitation learning, which passively learns from offline demonstrations, IIL allows human teachers to actively refine policies by correcting mistakes as they occur during execution. In general, IIL is a closed-loop learning paradigm in which a robot repeatedly executes its current manipulation policy and receives real-time human guidance to correct or refine its behavior. As illustrated in Figure 1, the overall process can be divided into two phases. The offline initialization phase comprises collecting expert demonstrations and training an initial policy—steps that follow the standard imitation-learning pipeline. The resulting pretrained policy then enters the interactive refinement loop, which characterizes IIL. In this loop, the policy is deployed on the robot, after which a human teacher monitors the robot’s behavior and provides on-policy feedback when errors, suboptimal actions, or failure cases occur. This feedback—ranging from corrective demonstrations to evaluative signals—is aggregated with existing datasets and subsequently used to retrain and update the policy. The loop iterates until the robot achieves the desired level of performance, enabling continual improvement under real-world execution conditions (Kelly et al., 2019; Celemin et al., 2022). In IIL for dexterous manipulation, the general concept of the refinement loop stays the same, but the underlying policy representations and the types of feedback a human provides have to adapt to the unique challenges of dexterous manipulation.

In literature, incorporating human interventions in training is also referred to as a human-in-the-loop approach (Celemin et al., 2022; Mandlekar et al., 2020; Wang C. et al., 2024). This human-in-the-loop approach ensures adaptability, enhances sample efficiency, and mitigates covariate shift by dynamically guiding the learning process. It is essential to note that, in our context, the interactive component of IIL refers explicitly to human feedback, rather than interactions with the environment, which may also be encompassed by broader interpretations of interactivity in learning. Hence, we believe IIL is promising for real-world dexterous manipulation applications.

While prior surveys cover different topics of robot manipulation like embodied learning (Zheng et al., 2025), imitation learning for dexterous manipulation (An et al., 2025), diffusion models in robotics (Wolf et al., 2025), in-hand manipulation (Weinberg et al., 2024), broader dexterous surveys (Li et al., 2025), and general IIL in robotics (Celemin et al., 2022), none provide a dedicated examination of IIL in the high-dimensional action space of multi-finger, contact-rich manipulation. Our survey fills this gap by identifying the specific challenges posed by contact-rich, multi-fingered dexterous manipulation. It sheds light on how IIL can address sample efficiency, safety, and robustness in real-world settings.

**FIGURE 2:**
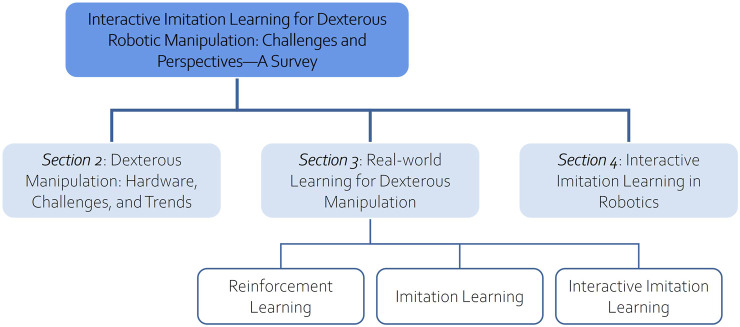
Structural overview of the survey paper on interactive imitation learning for dexterous robotic manipulation.

To give an intuitive understanding, we broaden our perspective to cover the two core dimensions highlighted above: Dexterous Manipulation and Interactive Imitation Learning. The organization of the paper is illustrated in Figure 2. Section 2 addresses the challenges and trends in dexterous manipulation, laying an essential groundwork for understanding this area of robotics. Section 3 delves into learning-based methods for real-world dexterous manipulation, exploring approaches from imitation learning and reinforcement learning to IIL. A broader examination of IIL in robotics, identifying its transferability to dexterous manipulation, is discussed in Section 4.

## 2 Dexterous manipulation: hardware, challenges, and trends

Dexterous Manipulation is a specialized field in robotics focused on controlling multi-fingered end effectors to grasp and manipulate objects effectively (Okamura et al., 2000). Anthropomorphic robot hands are designed to replicate human activities, offering great versatility. The most comprehensive models of the human hand typically incorporate 20–25 degrees of freedom (DOF), with each finger generally modeled with 4 DOF and the thumb with 4 or 5 DOF. Additionally, the palm and wrist are sometimes included with extra DOF to enhance the model’s fidelity (Zarzoura et al., 2019; Savescu et al., 2004). Despite their sophistication, anthropomorphic robotic hands face significant technical challenges, particularly in control accuracy, sensor and actuator system dimensioning, and the transmission of power and signals. Alternatively, a minimalist approach can address many of these issues using underactuated hands. These hands have fewer actuators than joints, relying on passive mechanisms like springs or tendons to simplify control and adapt to different object shapes (Birglen et al., 2008). Due to the inherent technical and control complexities, the availability of commercially produced anthropomorphic robotic hands was previously limited. Table 1 provides an overview of commercially available anthropomorphic robotic hands. Among them, 2/3 are nearly fully actuated, offering a level of dexterity that closely resembles human capabilities. This represents an increase compared to previous years, reflecting the rapidly evolving landscape of dexterous manipulation technologies. In addition to the hands listed, several companies—such as Figure AI^1^, 1X^2^, and Tesla^3^—are actively developing proprietary robotic hands as part of their humanoid platforms. However, these designs are not yet openly available for research or third-party development.

**TABLE 1:** Commercial anthropomorphic robotic hands.

Name	DoA	DoF	Actuation	No. of fingers	Fingertip force	Payload	Weight	Tactile sensors
Shadow dexterous hand (Shadow Robot Company, 2024)	20	24	Tendon-driven	5	—	5 kg	4.3 kg	Yes
TESOLLO DG-5F (TESOLLO, 2025)	20	20	Direct-drive	5	—	2.5–10 kg	1.7 kg	Yes
Agile hand (AGILE ROBOTS, 2023)	16	20	Mechanical	5	10 N	—	1.5 kg	No
ARTUS lite (Sarcomere Dynamics, 2024)	16	20	Tendon-driven	5	1.5 kg	5 kg	1.1 kg	No
Mimic hand (mimic robotics AG, 2024; Toshimitsu et al., 2023)	16	20	Tendon-driven	5	—	7 kg	1.1 kg	No
Unitree Dex5-1 (Unitree, 2025)	16	20	Mechanical	5	10 N	3.5–4.5 kg	1.0 kg	Yes
Allegro hand V4/V5 (WONIK ROBOTICS, 2023)	16	16	Direct drive	4	—	5 kg/15 kg	1.0 kg	Yes
LEAP hand (Shaw et al., 2023a)	16	16	Direct drive	4	—	—	—	No
PaXini DexH13GEN2 (PaXini, 2025)	13	16	Direct drive	4	15 N	5 kg	—	Yes
XHAND1 (ROBOTERA, 2025)	12	12	Direct drive	5	15 N	16–25 kg	1.1 kg	Yes
Schunk SVH (SCHUNK, 2023)	9	20	Mechanical	5	—	0.85 kg	1.3 kg	No
RH8D robot hand (Seed Robotics, 2021)	8	19	Tendon-driven	5	—	1–2.5 kg	620 g	Yes
RH56BFX/RH56DFX (INSPIRE-ROBOTS, 2024)	6	12	Mechanical	5	4 N/10 N	—	540 g	No
IH2 azzurra hand (PRENSILIA, 2023)	5	11	Tendon-driven	5	7 N	—	640 g	No
qb SoftHand2 research (qbrobotics, 2022)	2	19	Tendon-driven	5	—	2–3 kg	940 g	No

Degrees of Actuation (DoA), Degrees of Freedom (DoF), Payload depends on measurement method, tactile sensors include configurable options.

Robotic hands use different actuation methods to control movement, primarily mechanical links, tendon-driven, and direct drive. Mechanical links use rigid components like gears and levers to transmit force, offering precision but at the cost of bulkiness and reduced flexibility. Tendon-driven systems resemble human anatomy by using flexible tendons or cables to control joints, providing lightweight, adaptive, and fluid movements, though they require careful maintenance and calibration. Direct drive places actuators directly at each joint, ensuring highly accurate control with minimal mechanical play, but can be heavy and power-intensive, making it less suitable for compact designs. Each method has trade-offs between precision, adaptability, and complexity (Melchiorri and Kaneko, 2016).

While an underactuated robotic hand is generally sufficient for basic tasks, such as picking up and placing household objects (Groß et al., 2024), more intricate operations, such as in-hand manipulation or handling small objects, demand a robotic hand with enhanced agility. For example, an impressive demonstration of in-hand manipulation was presented by Akkaya et al. (2019), where one Shadow Dexterous Hand solved a Rubik’s Cube entirely within its grasp.

With the growing availability of advanced hardware and progress in learning-based control algorithms, dexterous manipulation has become an increasingly active area of research. Bibliographic searches in major databases such as Scopus^4^ and IEEEXplore^5^ reveal a wide range of topics within this field. Most research focuses on machine learning algorithms and the design of dexterous manipulators. In contrast, haptic and tactile interfaces receive comparatively less attention, despite their potential to enhance human-robot interaction (Li et al., 2025; An et al., 2025; Kadalagere Sampath et al., 2023; Yu and Wang, 2022; Li et al., 2022). Tactile feedback is essential for fine motor tasks and for perceiving object properties (Jin et al., 2023; Dahiya et al., 2010).

Research on machine learning algorithms for dexterous manipulation primarily includes grasp synthesis and manipulation skill/policy learning approaches. Grasp generation approaches utilize mainly classical neural networks (Blättner et al., 2023), Conditional VAriational Auto-Encoder (CVAE) (Zhao et al., 2024), normalizing flow (Feng et al., 2024) or reinforcement learning (Osa et al., 2018). Cluttered scenes represent one clear challenge in grasp generation (Blättner et al., 2023), where grasping planning also needs to consider other objects and uncertain observations, e.g., occlusions or partial observations of objects (Chen et al., 2024; Hidalgo-Carvajal et al., 2023; de Farias et al., 2024). Policy learning for dexterous manipulation is dominated by reinforcement learning and imitation learning approaches (Li L. et al., 2024; Wang D. et al., 2024; Han et al., 2024; Ze et al., 2023; Li et al., 2023). Human-in-the-loop approaches are particularly beneficial in policy learning, as this domain involves long-term decision-making where interactive feedback can significantly shape behavior over time (Liu et al., 2024; Chisari et al., 2022). In contrast, grasp generation is typically a single-step task, making it less suited for effective human guidance. Therefore, this survey focuses on policy learning in real-world environments, where human input can have the most significant impact.

## 3 Real-world learning for dexterous manipulation

Real-world learning for robots is challenging due to the high cost of training data, especially for high DOF robots and tasks. Integrating human prior knowledge can accelerate autonomous robot learning (Rayyes et al., 2023; Rayyes, 2021). We will survey how previous work has dealt with dexterous manipulation using imitation learning, reinforcement learning, and Interactive Imitation Learning.

### 3.1 Imitation learning

Imitation learning has emerged as a powerful tool in dexterous manipulation, enabling robotic systems to perform complex tasks by learning from human demonstrations. The versatility of imitation learning is showcased in a wide array of applications where robots are required to replicate human movements. However, the applications of dexterous manipulation using imitation learning are currently confined to relatively simple tasks. For instance, in a work by Amor in 2012, the focus was on the grasping of different mugs (Ben Amor et al., 2012). A more recent investigation by Ruppel expanded the scope to include pick-and-place operations, wiping tasks, and opening bottles using the Shadow Dexterous Hand (Ruppel and Zhang, 2020). Yi evaluated the grasping abilities of an Allegro Hand, testing it on ten different objects in simulation and five objects in a real-world environment (Yi et al., 2022). Arunachalam’s work, titled DIME, explored manipulation tasks such as flipping a rectangular object, spinning a valve, and rotating a cube on the palm using an Allegro Hand (Arunachalam et al., 2023b). Moreover, the Holo-Dex extension incorporated tasks like card sliding and can spinning (Arunachalam et al., 2023a). Publications that deal with long-horizon tasks specifically for dexterous manipulation are very rare. DexSkills is an exception (Mao et al., 2024). It supports the hierarchical construction of long-horizon tasks composed of primitive skills. In experiments, 20 primitive skills were used to create and execute various long-horizon tasks. For example, lifting and moving a box object with the Allegro Hand (Mao et al., 2024). Recent work with diffusion policies performed experiments on more complex manipulation tasks like wrapping plasticine, making dumpling pleats, and pouring (Ze et al., 2024). However, all those applications do not fully utilize the dexterity of an anthropomorphic hand. There is still significant potential in leveraging the multi-dimensional capabilities of robot hands for complex human-like manipulation, including using tools made for humans to extend the field of application.

While imitation learning has demonstrated promising results in dexterous manipulation, these achievements are closely tied to the specific methods employed. To understand how such outcomes are realized, it is essential to examine the diverse approaches developed to overcome the key challenges inherent to this domain. The following sections present a range of works that address three central challenges posed by dexterous manipulation: managing high-dimensional action spaces, handling multi-modal contact interactions, and enabling long-horizon task execution.

#### 3.1.1 The high-dimensional action space

The biggest challenge in dexterous manipulation lies in learning a policy capable of managing the high complexity required to control such a large action space effectively. It is known from statistical learning that the amount of training data generally increases with the complexity of the model (Hastie et al., 2009; Sutton and Barto, 2018; Lu et al., 2024; Kubus et al., 2018). It is, therefore, crucial to select a sample-efficient method to learn the policy with a manageable amount of data. However, this results in a trade-off with the policy’s reduced generalizability and dexterity, limiting its applications. Potential sample-efficient methods include splitting the policy into a learned and hard-coded part (Yi et al., 2022), reducing the action space to a low-dimensional latent space (Ben Amor et al., 2012; Hu et al., 2022; Liconti et al., 2024; He and Ciocarlie, 2022), reducing the input dimensionality of the policy in visual imitation learning (Ruppel and Zhang, 2020; Cai et al., 2024), or using non-parametric policies without learnable parameters (Arunachalam et al., 2023b; a).

An example of splitting the policy into two parts was presented in Yi et al. (2022) for grasping different objects. One part of the policy takes care of reaching the objects. As this part only considers the movement of the robot arm, it is fundamentally not different from a task with a non-dexterous gripper. Therefore, classical methods like Dynamic Movement Primitive (DMP) can learn this part of the policy efficiently with a small number of demonstrations (Saveriano et al., 2023). The second part consists of the actual grasping of an object, which includes identifying the object, predicting the object pose, and selecting a predefined grasping pose for the fingers. The finger motion planning is executed through a predefined strategy, as learning these finger trajectories via DMP has resulted in poor performance due to the high dimensionality.

Reducing the action space into a low-dimensional latent grasp space was inspired by how humans control their hands. Research studies show that the individual muscles in the hand are not controlled individually. Instead, the fingers are controlled by hand synergies (Santello et al., 2016; Starke and Asfour, 2024; He and Ciocarlie, 2022). These hand synergies can be modeled as a projection of the configuration space of the hand into a low-dimensional space. For instance, Ben Amor et al. (2012) utilizes the principal component analysis (PCA) for this projection, where the first component corresponds to the opening and closing of the hand, and higher-order components are used for more detailed hand motions. Ben Amor et al. (2012) states that only five dimensions are required to represent the relevant grasp movements. Using this low-dimensional grasp space, a DMP can be used to define the policy for the finger movement and a separate DMP for the wrist pose. The policy output is then mapped back to the original high-dimensional action space afterward. While PCA represents a linear mapping, a Variational Autoencoder (VAE) can learn a more complex mapping from the high-dimensional action space to a low-dimensional latent space from task-agnostic datasets, reducing the amount of expensive, task-specific training data in BC (Liconti et al., 2024).

Rather than simplifying the action space, some approaches focus on reducing the dimensionality of the policy’s input. This is particularly relevant—and increasingly common—in visual imitation learning, where policies are conditioned on high-dimensional image data (Nair et al., 2022). While not exclusive to dexterous manipulation, these techniques play a significant role in making policy learning more tractable in visually rich environments (Liu Q. et al., 2025). In Ruppel and Zhang (2020), the manipulated objects and the robot hand are represented as point sets. Feed-forward and recurrent policy networks are trained using this representation, which is constructed either by manually attaching LEDs to track positions or by using a CNN to generate virtual keypoints. Similarly, Gao et al. (2023) and Gao et al. (2024) use visual keypoints and geometric constraints to learn movement primitives, forming an object-centric task representation. The Multifeature Implicit Model (MIMO) (Cai et al., 2024) introduces a novel object representation that incorporates multiple spatial features between a point and an object. This approach enhances the performance of visual imitation learning for task-oriented object grasping with a robot hand.

While the previous methods use a parameterized policy in the form of DMP or neural networks, whose weights must be learned, non-parametric approaches derive the action to be executed directly from the training data. No time-intensive training on the policy is required. DIME (Arunachalam et al., 2023b) is an example. Here, state-based or image-based observations are utilized to find matches in the demonstrations via the nearest neighbor method to extract the following action. For image-based observations, dimensionality reduction with Bootstrap Your Own Latent (BYOL) (Grill et al., 2020) is performed before applying the nearest neighbor algorithm in the embedding space to find the action.

Still, several challenges and limitations hinder the current approaches to imitation learning for dexterous manipulation. When representing objects as point sets, only relying on a limited number of point markers from a motion tracking system as observations restricts the agent from supporting higher-dimensional observations like point clouds or images, which are necessary for more precise manipulation tasks (Ruppel and Zhang, 2020). Relying on predefined grasps for each object limits the system’s effectiveness when dealing with unknown objects (Yi et al., 2022), which is essential when expanding the application scope from a structured environment to an open world, such as households. Non-parametric approaches suffer from low success rates and low generalization in task situations where the visual complexity of the input can not be adequately encoded in latent space (Arunachalam et al., 2023b; a). Enlarging the latent space, on the other hand, requires more data to learn the encoder. The dimensionality reduction of the action space via principal component analysis results in low generalization to new tasks (Ben Amor et al., 2012). Due to the information loss, the dexterity of hand motions is reduced.

#### 3.1.2 Multi-modality due to contact interactions

Dexterous manipulation tasks often involve contact-rich interactions with the environment, leading to inherently multimodal action distributions—for example, multiple valid grasps or contact sequences that achieve the same goal. Naively averaging over such demonstrations, as in standard behavior cloning, often yields unnatural or ineffective actions. To address this, the key idea is to use probabilistic or structured policies that can represent a distribution over actions rather than a single deterministic output. Over the past few years, multiple solutions and combinations of those have been presented, primarily for non-dexterous tasks (Urain et al., 2024):

- Latent variable/Sampling Models—such as VAEs, GANs, and Normalizing Flows—address multi-modality by explicitly modeling distributions over actions through a latent space conditioned on context. These models enable efficient sample generation and capture diverse behavioral modes, making them well—suited for representing the inherent variability in contact-rich manipulation tasks. One popular example of this category is the Action Chunking with Transformer (ACT) algorithm (Zhao et al., 2023).
- Mixture Density Models (MDMs) represent action distributions as weighted combinations of parametric densities—typically Gaussians—conditioned on contextual inputs. By modeling multiple modes explicitly, MDMs provide a principled approach to capturing multi-modality in continuous action spaces (Shafiullah et al., 2022; Zhu et al., 2023; Mees et al., 2022).
- Energy-Based Models (EBMs) define action distributions implicitly via an energy function, where low-energy regions correspond to high-probability behaviors. Sampling typically requires iterative optimization or stochastic methods such as Langevin dynamics (Florence et al., 2022).
- Discretized Action Models/Categorical models approximate continuous action spaces by discretizing them into a finite set of tokens, enabling the use of classification-based generative architectures. These models capture multi-modality by representing action distributions as categorical probabilities over discrete tokens, often leveraging spatial value maps or autoregressive structures to model complex, high-dimensional behaviors (Shafiullah et al., 2022; Brohan et al., 2023; Zitkovich et al., 2023).
- Diffusion Models (DMs) generate samples through an iterative denoising process that transforms noise into structured data, effectively modeling complex distributions over actions. By parameterizing the score function of an implicit energy landscape, DMs capture multi-modality via successive refinements of noisy inputs, enabling expressive and composable generative modeling despite slower inference compared to direct sampling approaches (Chi et al., 2024a; Reuss et al., 2024; Wolf et al., 2025; Freiberg et al., 2025).

Diffusion models, in particular, have gained popularity for modeling multimodal distributions in high-dimensional action spaces, making them well-suited for dexterous tasks requiring fine-grained contact reasoning. Their ability to generate diverse behaviors that reflect real-world stochasticity comes at the cost of increased computational demands and reduced sample efficiency. For example, state-of-the-art diffusion policies often require over 100 expert demonstrations to achieve proficiency (Chi et al., 2024a; b; Pearce et al., 2023). An exception is the 3D Diffusion Policy (3DP) (Ze et al., 2024), which enhances training efficiency by encoding sparse point clouds into compact 3D representations using a lightweight MLP encoder. Conditioning on these compact representations—rather than raw sensory inputs—accelerates learning and improves generalization, enabling successful policy training with as few as 10–40 demonstrations across both simulated and real-world dexterous manipulation tasks. Notably, omitting color information from point clouds further increases robustness to novel objects. Building on 3DP, FlowPolicy (Zhang et al., 2025) introduces an extension that significantly improves inference speed. By employing consistency flow matching, it enables action generation from noise in a single inference step, while maintaining comparable success rates.

The diversity of approaches for modeling multi-modal action distributions reflects the complexity of contact-rich manipulation tasks. Each class of policies offers distinct trade-offs. Latent variable and mixture density policies are lightweight and support efficient sampling, but may struggle with highly complex or discontinuous behaviors. Energy-based and diffusion policies provide greater expressiveness and compositionality but incur slower inference and higher data requirements. Diffusion methods can be viewed as conceptually bridging EBMs and latent-variable approaches: they exploit score-based training to navigate implicit energy landscapes while maintaining a structured generative process. Yet their computational demands and reliance on large datasets limit applicability in low-data or real-time settings. Discretized action policies provide a pragmatic alternative by converting continuous control into sequence prediction, allowing the use of powerful architectures like transformers. Their performance, however, depends on discretization granularity and can suffer from reduced precision in fine motor tasks.

#### 3.1.3 Performing long-horizon tasks

Policies for long-horizon tasks are primarily formulated at a higher level of abstraction and do not consider the low-level control of individual joints. This area of research is often referred to as task planning (Russell and Norvig, 2016; Guo et al., 2023). Here, imitation learning is also popular in learning task plans from human teachers (Diehl et al., 2021; Ramirez-Amaro et al., 2017). Although research in dexterous manipulation mainly focuses on low-level policies for short-horizon tasks, some approaches combine both levels, such as DexSkills (Mao et al., 2024). DexSkills views a robot’s task from a hierarchical perspective to be able to execute long-horizon tasks. The core idea is to decompose complex tasks into primitive skills. The system includes several key components. First, a temporal autoencoder extracts a latent feature space from the demonstrations. Instead of images, the observation consists of the robot’s joint states, tactile information, and contact status. Second, a label decoder segments a task into primitive skills based on the latent representation. Finally, a multilayer perceptron (MLP) learns the state-action pairs for each primitive skill separately from human demonstrations via BC. After the initial training, new long-horizon tasks can be learned from a single demonstration. The primitive skill sequence is extracted via the label decoder and autonomously executed by the robot using the provided label sequence. Although DexSkills is promising, its’ reliance on predefined primitive skills limits the range of possible long-horizon tasks. Additionally, segmenting demonstrations into individual primitive skills requires supervised training with labeled data, making data acquisition expensive despite the relatively small amount of training data needed (Mao et al., 2024).

To address these challenges, recent research has begun exploring the integration of Vision-Language Models (VLMs) into imitation learning pipelines. Approaches such as RoboDexVLM (Liu H. et al., 2025) and DexGraspVLA (Zhong et al., 2025) leverage the generalization capabilities of large-scale pretrained models to interpret high-level task descriptions and infer action sequences from diverse visual inputs, including third-person videos. These models offer a way to bridge the embodiment and viewpoint gap by grounding language in visual context and enabling robots to reason about tasks in a more abstract, flexible manner. By combining the structured decomposition of tasks—as seen in hierarchical methods like DexSkills—with the broad priors and multimodal understanding of VLMs, these approaches promise more scalable and generalizable solutions for long-horizon dexterous manipulation. But with the use of VLMs for task planning and reasoning, the inference time increases significantly, which limits real-time deployment. Additionally, the predefined primitive skills impose a ceiling on the generalization capabilities of such methods.

Another complementary direction is leveraging large-scale human demonstration videos—especially those sourced from the internet—as a means to improve data diversity and reduce the cost and effort of expert data collection (Shaw et al., 2023b; 2024; Mandikal and Grauman, 2022; Sivakumar et al., 2022; Qin et al., 2022b). Such videos offer a rich prior over manipulation behaviors and object interactions, which can support better generalization across tasks and environments. However, bridging the domain gap between third-person human video and robot execution, particularly with dexterous hands, introduces substantial challenges. These include differences in embodiment (e.g., human hands vs. robot hands), viewpoint and occlusion issues, the lack of action labels, and the difficulty of inferring precise 3D contact-rich motions from 2D videos.

The limitations discussed above underscore the need for more robust and flexible approaches to advance imitation learning in dexterous manipulation. The highlighted publications reveal two most prominent challenges: generalization across tasks and the ability to handle long-horizon behaviors. Methods such as DexSkills, vision-language models (VLMs), and 3D diffusion policies offer promising directions to address these issues. A particularly compelling avenue for future research lies in combining hierarchical skill composition, as demonstrated in DexSkills, with the generative flexibility of diffusion policies. Such integration could reduce overall training effort by enabling the reuse of learned skills across diverse tasks, thereby improving scalability and efficiency.

Still, with imitation learning approaches, major limitations remain. In addition to the covariant shift problem, imitation learning approaches that have learned behavior in a supervised mode have the disadvantage that the quality of the demonstrations limits the performance of the resulting policy. Consequently, demonstrations represent an upper bound that the policy cannot exceed. To improve the policy beyond the level of the demonstrations, a criterion must be defined that can be used to measure and optimize performance. This problem is addressed in reinforcement learning, in which a reward function is defined that provides the agent with feedback on its performance. Based on this feedback, the agent can optimize its behavior and thus improve its performance. In the context of reinforcement learning, a policy is learned by the agent through numerous interactions with the environment. The data collected in the process is used to train the policy. The requirement for extensive interaction poses a significant challenge when training a robot using reinforcement learning in the real world. Firstly, the large number of trials can be very time-consuming, and human intervention is required to reset the environment between episodes. Secondly, the actions the agent selects may be hazardous or cause physical damage to the hardware, posing risks to both the robot and its surroundings and limiting their real-world learning. However, some approaches allow exploiting the potential of reinforcement learning on physical robots, including dexterous manipulation. These will be discussed in more detail in the next section.

### 3.2 Reinforcement learning

Using reinforcement learning for real-world dexterous manipulation is categorized in three directions in the literature (Yu and Wang, 2022). The classical reinforcement learning approach involves learning from scratch, in which a novice agent learns purely by interacting with a physical robot and the environment. The second approach uses a pre-trained agent to begin learning from a safer and more informed initial policy, typically achieved by combining reinforcement learning with imitation learning. Lastly, the agent is trained in simulation, and then the policy is transferred to the physical robot. Some relevant examples of those approaches are presented below.

#### 3.2.1 Reinforcement learning from scratch

Learning from scratch is mainly done in simulation, as data generation is significantly more cost-effective. Implementing this approach directly on a physical robot poses additional challenges beyond those already mentioned, particularly the difficulty of accurately retrieving the representation of the environment’s state. Perceiving the environment is typically achieved using vision-based sensors, such as cameras (Haarnoja et al., 2018; Luo et al., 2021). However, this approach comes with a trade-off in sample efficiency, as the high-dimensional data generated increases both computational demands and processing complexity. Instead, introducing tactile sensors in observation reduces the sample complexity for dexterous manipulation significantly (Melnik et al., 2019). van Hoof et al. (2015) uses pure tactile information while Falco et al. (2018) combines tactile and visual observations in policy learning with reinforcement learning for an in-hand manipulation task. To avoid costly resets of the environment by humans, Gupta et al. (2021) presents a reset-free approach, where learning multiple tasks simultaneously and sequencing them solves the problem automatically. The individual tasks provide the reset for other ones.

#### 3.2.2 Reinforcement learning and demonstrations

Combining reinforcement learning with imitation learning overcomes issues of both approaches (Nair et al., 2018; Hester et al., 2018). On the one hand, the sample complexity is significantly reduced by using demonstrations to pre-train a policy. On the other hand, through reinforcement learning, the robot can obtain further information through interaction with the environment and fine-tune its performance beyond the quality of demonstrations. Nevertheless, especially in the field of dexterous manipulation, many approaches still rely on training and experiments in simulation due to efficiency, safety, and cost reasons (Rajeswaran et al., 2018; Radosavovic et al., 2021; Huang et al., 2023; Mosbach et al., 2022; Qin et al., 2022b; Han et al., 2024; Mandikal and Grauman, 2022; Orbik et al., 2021). Only a few studies use reinforcement learning with demonstrations on physical robots (Gupta et al., 2016; Zhu et al., 2019; Nair et al., 2020). Their ability to conduct real-world experiments hinges on the constraint of basing their policy on low-dimensional state spaces, resulting in a lower-capacity policy that can be efficiently trained on less data.

Utilizing object-centric demonstrations—focusing solely on the manipulated object’s trajectory—has shown potential in training tasks for soft robotic hands, as illustrated in Gupta et al. (2016). Their approach is based on Guided Policy Search (GPS) (Levine and Abbeel, 2014), which offers advantages in learning high-dimensional tasks. Multiple policies are initially learned from demonstrations and refined using model-based reinforcement learning to follow the demonstrated behavior closely. These are then distilled into a single neural network policy via supervised learning. However, the final policy’s performance was limited, primarily due to the soft and compliant nature of the RBO Hand 2 robotic hand. More effective results have been achieved with the Demo Augmented Policy Gradient (DAPG) method introduced by Rajeswaran et al. (2018), which has become a widely adopted approach in dexterous manipulation (Qin et al., 2022b; Huang et al., 2023; Qin et al., 2022a). It combines model-free, on-policy reinforcement learning with imitation learning to enhance policy exploration and reduce sample complexity. Pre-training with BC equips the agent with an intuitive understanding of task-solving strategies before its autonomous exploration. Although initially tested in a simulated environment, DAPG’s applicability to real-world manipulation was later validated by Zhu et al. (2019) using an Allegro Hand. Demonstrations were shown to significantly accelerate training in the real world—cutting training time from 4 to 7 h without demonstrations to 2–3 h with 20 demonstrations. Another approach, the Advantage-Weighted Actor-Critic (AWAC) algorithm introduced by Nair et al. (2020), combines offline reinforcement learning with online fine-tuning using off-policy methods. Off-policy algorithms are generally more sample-efficient, as they can reuse offline data during the online learning phase. AWAC formulates the policy improvement step as a constrained optimization problem, ensuring that the updated policy remains close to the expert demonstrations. Its effectiveness is demonstrated on an object-repositioning task using a four-fingered dexterous robotic hand. The authors also note that tuning AWAC’s parameters can be challenging. A direct comparison between DAPG and AWAC on dexterous manipulation tasks shows that AWAC can achieve faster learning and better data efficiency than DAPG (Nair et al., 2020).

#### 3.2.3 Sim-to-real reinforcement learning

While some studies have successfully implemented reinforcement learning directly on physical robots, with or without demonstration, simulation remains the most practical and widely utilized environment for training due to its cost-effectiveness, scalability, and safety. The complete freedom concerning safety restrictions and the almost unlimited data availability through parallelization offer a unique starting point for reinforcement learning. Still, sim-to-real zero-shot achieves only limited performance due to the reality gap (Gilles et al., 2024; 2025). Especially for dexterous manipulation with its complex dynamics, it is challenging to create a simulation model that corresponds to reality. Developing more powerful and realistic simulators like Isaac Sim^6^, GENESIS^7^, and MuJoCo (Todorov et al., 2012) will not completely close the reality gap. A common method to overcome this gap is domain randomization, where the simulation is randomized with disturbances to compensate for inaccuracies in the modeling (Kumar et al., 2019; Akkaya et al., 2019; Andrychowicz et al., 2020). This can include the randomization of light, textures, or friction parameters. Randomization facilitates the agent’s adaptation to a wide range of environments, where the real world might represent one instance of this spectrum. Training a single robotic hand to solve a Rubik’s cube shows how powerful sim-to-real methods can be (Akkaya et al., 2019), but also how much computational resources are required to train such a complex policy: over 900 parallel workers were used over multiple months to collect data corresponding to 13.000 years of experience in simulation. We believe that using such a vast amount of computational resources to train a single task is not an effective approach. Instead, it may be more reasonable to leverage simulators and computational resources to pretrain a comprehensive foundation model with general knowledge—for example, about physical properties of the world—so that task-specific knowledge can subsequently be fine-tuned more efficiently. This was done, for example, by NVIDIA, when training the Generalist Robot 00 Technogly (GR00T) model on synthetic data from simulators and also real world data (NVIDIA et al., 2025).

While approaches based on reinforcement learning have shown impressive results in dexterous manipulation, particularly by utilizing sim-to-real methods for transferring policies from simulation to physical robots, they still face significant limitations. Data efficiency and safety remain key challenges, even when using demonstrations for a warm start. Additionally, the reliance on sim-to-real methods limits the system’s ability to adapt effectively to unstructured and highly dynamic environments.

### 3.3 Interactive imitation learning for dexterous manipulation

As outlined in the introduction, integrating humans interactively into the learning process represents a promising research direction. This approach helps mitigate challenges in imitation learning, such as covariate shift, and reduces the effort required for collecting demonstrations. Despite its potential, only a limited number of works have explored interactive human involvement in real-world dexterous manipulation tasks (Kaya and Oztop, 2018; Argall et al., 2011; Ugur et al., 2011; Sauser et al., 2012; Ding et al., 2023; Si et al., 2024; Wang C. et al., 2024). Not all of these works aim to learn generalized policies. Some intersect with shared control schemes (Kaya and Oztop, 2018), while others focus on identifying graspable regions of objects (Ugur et al., 2011). Among those that do learn policies, the objectives vary: learning stable grasps (Sauser et al., 2012), grasping strategies (Argall et al., 2011), human-like motion characteristics (Ding et al., 2023), or complex dexterous manipulation skills (Wang C. et al., 2024; Si et al., 2024).

The following section presents these works to provide an overview of the field and to highlight how they differ from the understanding of Interactive Imitation Learning adopted in this survey. The limited number of publications suggests that IIL has yet to gain widespread traction in dexterous manipulation, though recent contributions offer promising starting points.

In Kaya and Oztop (2018), an in-hand manipulation is learned interactively using a 16-DoF robotic hand. The task consists of swapping the position of two balls in the hand using skillful finger and hand movements. The human operator learns to control the robot arm via kinesthetic teaching while the hand runs a periodic finger movement. After the human operator has learned how to control the robot, successful executions of human and robot control are combined into a fully autonomous policy via regular imitation learning with a DMP. Although the human operator interacts with the robot during execution, this is not interactive imitation learning; instead, the interactive part belongs to the shared control scheme (Abbink et al., 2018), as the policy is learned after all data have been collected, like in normal imitation learning.

In Ugur et al. (2011), a 16-DoF robotic hand is used to grasp and lift various objects. The learning is supported by a human teacher and called parental scaffolding. Initially, the robot performs rough reach motions towards the object’s center. The human teacher can interfere by physical contact with the robot’s motion to achieve successful grasping. The robot checks the distance between its fingers and the object and records “first-touch” points on the object in case of contact, which correspond to graspable parts of the objects. A classifier that differentiates whether a voxel is graspable is trained using these “first-touch” points. The classifier is based on a newly proposed metric that captures the relationship between graspable voxels and all voxels of the object. The main focus lies on learning and inferring graspable parts of the objects, not the motion of grasping the object itself. Therefore, only a simple lookup table-based mechanism is used to select a reach-grasp-lift execution trajectory to grasp an object.

An early interactive imitation learning related work that learns a manipulation policy is presented in Argall et al. (2011). It introduces the Tactile Policy Correction (TPC) algorithm to learn how to grasp simple objects with a dexterous 8-DoF robotic hand from human interactions. The approach consists of two phases. First, a dataset of human demonstrations is created through teleoperation, allowing the robot agent to derive an initial policy via BC. In the second phase, the agent executes the policy and receives corrective tactile feedback from the human teacher to adapt the policy. The teacher physically touches the robot on five touchpads on the arm to provide feedback. Policy execution consists of object pose prediction and action selection. It utilizes GMM-GMR: Gaussian Mixture Model (GMM) encodes demonstrations, and Gaussian Mixture Regression (GMR) predicts target poses. The GMM is trained with weighted Expectation-Maximization (EM). In a subprocess, an inverse kinematic controller is responsible for selecting the appropriate action to reach the target pose. The policy undergoes adaptation through recurrent derivation from the updated dataset. This dataset evolves through tactile corrections from the human teacher, either through policy reuse, involving modification of existing data points, or refinement, entailing the addition of new data points. The presented experiments show that refinement is more effective than providing more demonstrations via teleoperation. A very similar approach is used to learn stable grasps for dexterous robot hands (Sauser et al., 2012). The grasping task is also modeled as GMM; the difference is in how the human interacts with the robot. The human teacher provides corrections by pressing on the fingertips to encourage better contact and shift the pose as much as possible within the compliance of the hand. The robot generates self-demonstrations by following the pose-pressure pair recorded from the previous step. This process extends the dataset by incorporating data that is free from the influence of correction forces, enabling more accurate and autonomous learning.

A different kind of human feedback is used in Ding et al. (2023) to train a policy for a dexterous hand. Instead of showing how to do the tasks, the feedback of the human evaluates the policy. The policy is learned via reinforcement learning in simulation and later fine-tuned with human feedback to enhance its human-like characteristics. Although no demonstrations are used to train the policy, and only human feedback in the form of preferences over generated trajectories is provided, this approach still exemplifies interactive learning for dexterous manipulation. The human feedback primarily aims to make the policy execution more human-like. The human teacher is presented with two generated trajectories, and the provided feedback is the choice of which trajectory is more human-like. Based on this feedback, a reward model is trained to fine-tune the policy via reinforcement learning later. For execution on a physical robot, no additional training is performed; instead, the policy is directly transferred to the physical robot showing its robustness against the reality gap.

With the recent advent of diffusion models, diffusion-based imitation learning approaches have gained significant popularity. These models are particularly well-suited for managing multimodal action spaces in dexterous manipulation. In the work titled Tilde (Si et al., 2024), a unique integration of diffusion-based imitation learning with DAgger-based on-policy updates is employed to perform dexterous manipulation tasks using a high DOF robotic hand. This novel combination leverages the strengths of both methods, where diffusion models handle complex, multimodal action spaces effectively, and DAgger (Ross et al., 2011) provides robust, real-time corrections through human teacher interventions in case of failures. The robotic hand, known as DeltaHand, features a non-anthropomorphic design with four fingers, each possessing 3 DoF. Demonstrations for seven distinct manipulation tasks are recorded using a kinematic twin teleoperation interface. A vision-conditioned diffusion policy is then learned, utilizing input from an in-hand camera and the joint states. Integrating on-policy expert corrections via DAgger helps mitigate covariate shifts, ensuring more reliable performance. However, the authors also note that generalization to unstructured environments is limited, and the movement of the robot arm for more complex tasks has not yet been considered. This is likely due to the direct conditioning of the policy on images and, therefore, the limited data used to train the policy. An approach to address this is DexCap (Wang C. et al., 2024), which introduces a portable hand motion capture system and an imitation learning framework. The motion capture system consists of a motion capture glove for finger tracking and three cameras for wrist tracking and environment perception. The portability allows the accessible collection of demonstration data for bimanual tasks. DexCap enables robots to learn bimanual dexterous manipulation from human motion capture data (i.e., human motion capture data) through a diffusion policy, using a point-cloud-based BC algorithm. The learning process consists of three steps: First, the motion capture data is retargeted into the robot’s operational space, which includes mapping the finger positions and 6-DOF wrist pose into the action space of the robot via inverse kinematics. Secondly, a diffusion policy is trained based on down-sampled colored point cloud and retargeted data. Using colored point clouds transformed into a consistent world frame as input for the policy, rather than RGBD images, the system maintains stable observations even when the camera moves. Lastly, human operators can intervene and provide on-policy corrections to correct unexpected robot behavior. Corrections can be supplied as residual actions on top of the policy’s actions or by taking complete control and guiding the robot via teleoperation. Corrections and original demonstrations are used together to refine the policy. Experiments demonstrate a 33% improvement by fine-tuning with corrections on six household manipulation tasks with two 16-DOF robotic hands.

The works presented in this section are among the few that incorporate interactive human feedback into the learning process for dexterous manipulation. Table 2 shows an overview of these works with the used policy representation and performed tasks. The limited number of such studies may be attributed to several factors. One major constraint is the need for physical hardware to enable safe and effective human-robot interaction and to evaluate learning outcomes—an especially costly requirement in the context of dexterous manipulation. Additionally, the availability of suitable robotic hands remains limited, further restricting experimentation. Beyond hardware, unlike domains where large, static datasets can be curated upfront, the usefulness of human feedback in IIL depends on the current policy’s performance, making dataset creation and reuse non-trivial. Integrating human feedback is also more complex in dexterous manipulation due to the high dimensionality of actions and the intricacies of contact-rich dynamics. Historically, research in this domain has focused heavily on reinforcement learning (RL) as the primary method for solving complex tasks. However, the practical limitations of RL—particularly its inefficiency and resource demands when applied to real-world hardware—have prompted a shift in perspective. As these limitations became more apparent, interest in interactive imitation learning (IIL) began to grow. In dexterous manipulation specifically, sim-to-real transfer remains a significant challenge, often requiring substantial engineering effort to bridge the gap between simulation and physical deployment. More recently, the rise of humanoid robots in industry (BMW AG, 2024; Reuters, 2024), which are increasingly being used in roles traditionally filled by humans and are expected to operate human tools, has further fueled interest in efficient training methods. In this context, IIL has emerged as a promising approach for improving learning efficiency in high-dimensional manipulation tasks.

**TABLE 2:** Overview of IIL works for dexterous manipulation based on policy representation and feedback type. Sorted by publication year.

Reference	PR	RW	CF	EF	CF+EF	Tasks
Lu et al. (2025)	DV	✓	●			Raise hand, packing, insertion
Si et al. (2024)	DG	✓	●			Grasp, block slide, block lift, ball roll, cap twist, syringe push, shape insert
Wang et al. (2024a)	DG	✓	●			Packing, scissor cutting, tea preparing
Ding et al. (2023)	CP	✓		●		Dexterous manipulation, pen, relocate, pushing
Luo et al. (2023)	DV			●		Adroit, locomotion
Gams et al. (2016)	CP	✓	●			Wiping surface
Sauser et al. (2012)	CP	✓	●			Grasping
Argall et al. (2011)	CP	✓	●			Grasping

Policy representation (PR), Real-world experiments (RW), Corrective feedback (CF), evaluative feedback (EF).

CP: Classical parametric - linear models, GMM, RBF, DMP/ProMP, shallow MLP.

DV: Deep vision-conditioned - CNN/LSTM that ingest images or point clouds.

DG: Diffusion/generative - score-based or flow models that explicitly model multimodality.

Despite its potential, the field remains largely unexplored. Current works demonstrate the viability of IIL, but also reveal gaps—such as the limited use of tactile feedback and generalization in unstructured environments—that point to valuable directions for future research. Exploring how IIL methods developed in other areas of robotic manipulation can be adapted to dexterous tasks may yield important insights and broaden the scope of innovation in this field. Therefore, we next provide an overview of IIL as an emerging paradigm, highlighting the still limited but rapidly growing body of work in this area.

## 4 Interactive imitation learning in robotics

While this survey has focused on dexterous manipulation thus far, this chapter examines approaches to the role of interactive imitation learning in general robotics, aiming to identify its potential for dexterous manipulation. We categorize the approaches in IIL into two directions, which differ in how humans provide real-time feedback to the agent.

1. Corrective feedback, where the human teacher gives feedback on *how to improve* the execution in the action domain. So either the human gives absolute actions that replace the agent’s actions (Kelly et al., 2019), or the human provides relative corrections (Celemin and Ruiz-del-Solar, 2019), guiding the agent toward correct actions.
2. Evaluative feedback, where the human teacher gives feedback on *how well* the agent performs (Bradley Knox and Stone, 2008). In this case, the human teacher does not need task expertise, but only the ability to evaluate performance. This feedback can be delivered as absolute evaluative signals—commonly referred to as human reinforcement—or as relative preferences between differentJ agent behaviors.

**FIGURE 3:**
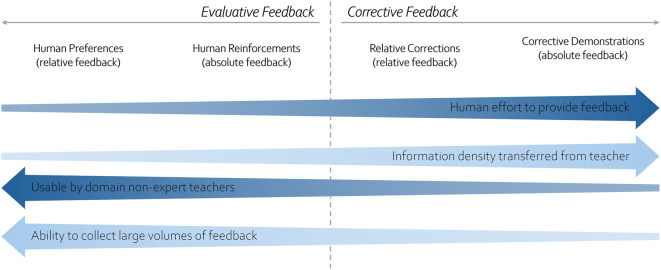
Comparison of Human Feedback Modalities, modified from Celemin et al. (2022).

The choice of feedback modality in a learning application often depends on the level of autonomy desired in the agent’s exploration process. For example, evaluative feedback typically provides only limited guidance on the optimal policy, requiring the agent to rely more heavily on autonomous exploration. In contrast, corrective feedback or demonstrations convey more direct information about the policy, increasing reliance on human supervision. This trade-off is commonly described in the literature as the exploration-control spectrum (Najar and Chetouani, 2021). Figure 3 summarizes common forms of human feedback, comparing them along key dimensions such as information content, human effort required, expertise needed, and scalability.

In IIL, various corrective feedback approaches have emerged from the original DAgger (Ross et al., 2011) approach and developed further. While DAgger was one of the first algorithms to describe the interactive intervention of an expert in training, the expert in DAgger usually consisted of another algorithmic expert since this expert had to relabel each data sample of the agent. This would be too much work for a human expert, at least in robotics. Nevertheless, many approaches based on DAgger have been developed. For example, LazyDAgger (Hoque et al., 2021), where an additional learned meta-controller in the agent decides whether an expert should be consulted, thus reducing the number of human interactions. Instead of the agent deciding when it needs expert support, in most current approaches, the human teacher decides when a correction is necessary, for example, if the agent enters unsafe areas of the state space. HG-DAgger (Kelly et al., 2019) is an algorithm where the human teacher (expert) chooses when to take over control. HG-DAgger is also used in the work RoboCopilot (Wu et al., 2025), which presents a complete bi-manual teleoperation system that allows seamless human takeover by using a leader-follower approach. The teleoperation device is a kinematic replica of the robot arm with a user interface especially designed for an interactive learning setting. While HG-DAgger involves only one expert who is as perfect as possible, MEGA-DAgger (Sun et al., 2023) allows for several experts who may also be imperfect. A built-in filter resolves contrary corrections and removes unsafe demonstrations. HG-DAgger only learns from the generated data when the expert takes control, so-called supervisor-generated data. This means that HG-DAgger learns how to recover from error situations but not how to stay within the target area. In addition, the policy changes substantially in each iteration when training only with supervisor-generated data and ignoring agent-generated data. Intervention Weighted Regression (IWR) (Mandlekar et al., 2020) addresses this problem by storing all the generated data. The agent-generated samples are stored in a separate dataset. Thus, one dataset has agent-generated data, and one has supervisor-generated data. During the policy training, an equal number of samples from both datasets are used to update the policy. This way, samples from interventions are weighted more heavily, with the idea that these samples are more likely to indicate bottlenecks in state space and thus be learned more robustly. Pang et al. (2025) takes a slightly different approach and proposes using agent-generated data that lead to errors as examples of “what not to do,” and using this data accordingly in their ergodic imitation learning framework, leading to more robust policies. Instead of providing corrections that push the task forward from out-of-distribution states, Hu et al. (2025) proposes a data-collection protocol that requires the teacher first to record recovery motions to return to familiar states, then record correction motions to finish the task from there.

Other approaches integrate corrective feedback with reinforcement learning to leverage the efficiency of corrective feedback while benefiting from reinforcement learning’s ability to optimize beyond experts’ performance. For example, Celemin et al. (2019) and Luo et al. (2021) propose learning a policy from supervisor-generated data (such as demonstrations and corrective feedback) while at the same time manually defining a reward function. This allows the agent to improve its policy continuously, achieving faster convergence than traditional reinforcement learning. However, shaping a reward function is a demanding engineering task. In another approach, Parnichkun et al. (2022) modifies the objective function of the reinforcement learning algorithm to incorporate the BC objective, directly aligning it with supervisor-generated data but continuously improving its policy with reinforcement learning.

IIL-algorithms utilizing evaluative feedback are a more direct way of bridging imitation and reinforcement learning. Many methods frame the robot control problem as a reinforcement learning task but impose the constraint that the environment lacks a predefined reward function. Instead, the agent’s performance is assessed through evaluative feedback provided by a human teacher. An early example of this approach is the TAMER framework (Bradley Knox and Stone, 2008), where a human teacher assigns scalar rewards based on its evaluation of the agent’s behavior. The agent’s objective is to select the action that maximizes this human-given reward for a given state. To achieve this, the agent learns a model of the human reward function using supervised learning techniques. Macglashan et al. (2017) proposes an alternative approach in which human feedback depends on the agent’s current policy maturity. He models human feedback as an advantage function, capturing the nuance that human teachers provide different evaluations depending on whether the agent is improving or performing adequately in the *status quo*. This more accurately reflects the dynamic nature of human feedback during the learning process. Although a reward function represents an evaluative feedback system in reinforcement learning, it can also be constructed by utilizing corrective feedback. For example, Luo et al. (2023) and Kahn et al. (2021) enable human teachers to give corrective feedback but only use it to indicate that an intervention occurred without considering the specifics of the intervention. These methods train an agent to minimize the probability of human intervention rather than focusing on the content of the feedback itself. This way, it’s robust to imperfect experts, as the expert can also provide wrong corrections, but only the fact that a correction happens helps the agent. Another way to deal with humans lacking expertise in a task is through evaluative feedback in the form of preferences between two presented executions. On the exploration-control spectrum, this approach leans towards autonomous exploration, similar to classical reinforcement learning. However, as Christiano et al. (2017) demonstrated, it can be applied to problems where a reward function cannot be explicitly defined. This method uses supervised learning techniques to infer a reward function from preference data. Since human preferences convey limited information, learning relies heavily on autonomous exploration, which often requires numerous and sometimes unsafe interactions between the agent and its environment. Consequently, this approach is primarily suited to simulated environments, making it less practical for real-world applications.

Combining corrective and evaluative feedback has produced promising results (Lu et al., 2025; Luijkx et al., 2025). For instance, in Spencer et al. (2020), in addition to recording corrected actions, each sample is tagged with a flag indicating whether the robot’s current state is good enough. States without expert intervention are assumed to be acceptable. This state evaluation allows the agent to learn a value function in parallel, enabling it to refine its policy even without further input from the expert, as the current execution is generally considered good enough. However, human experts cannot provide corrections in every situation. The work “Correct me if I am wrong” (Chisari et al., 2022) addresses this issue by extending IWR (Mandlekar et al., 2020) to handle cases where no correction is possible. In such situations, evaluative feedback allows the expert to discard state-action pairs that might otherwise pollute the training data. In the Active Skill-level Data Aggregation (ASkDAgger) framework (Luijkx et al., 2025), the human teacher can provide feedback in three ways: validation, relabeling, and annotation demonstrations, which represent a combination of evaluative and corrective feedback. Its FIER (Foresight Interactive Experience Replay) module integrates this validation, annotation, and relabeling feedback into the demonstration dataset. The PIER (Prioritized Interactive Experience Replay) module prioritizes using these mixed evaluative-and-corrective demonstrations during training–allowing the novice to learn both what not to do and what to do more efficiently.

Not only the feedback type is a relevant categorization criterion for IIL approaches, but also the different types of policy representations used are interesting. However, when examining different methods of policy representation, it becomes clear that the choice of representation, as in other robotic applications, depends heavily on the specific task rather than being unique to IIL. A variety of function approximators for policy representation are used in IIL, including linear models, radial basis functions (RBFs) (Celemin and Ruiz-del-Solar, 2019), classical feed-forward neural networks (FFNs) (Kelly et al., 2019; Sun et al., 2023), convolutional neural networks (CNNs) (Pérez-Dattari et al., 2020; Hoque et al., 2021), recurrent neural networks (RNNs)/long short-term memory networks (LSTMs) (Mandlekar et al., 2020; Chisari et al., 2022), diffusion models (Si et al., 2024; Wang C. et al., 2024), as well as DMPs (Gams et al., 2016; Celemin et al., 2019) and Probabilistic Movement Primitive (ProMP) (Ewerton et al., 2016). An overview of recent IIL works, their policy representations, feedback types, and tasks is presented in Table 3.

**TABLE 3:** Overview of IIL works based on policy representation and feedback type. Sorted by publication year.

Reference	PR	RW	CF	EF	CF+EF	Tasks
Hu et al. (2025)	DG	✓	●			Long horizon shirt hanging, lid sealing, box packing, assembly
Knauer et al. (2025)	CP	✓	●			Pick and place
Lee et al. (2025)	DG	✓	●			Stacking, pushing, plugging, loading
Luijkx et al. (2025)	DV	✓			●	Packing, assembly
Luo et al. (2025)	DV	✓	●			Motherboard assembly, furniture assembly, belt installation, jenga
Pan et al. (2025)	DG	✓	●			Raspberry harvesting, cloth wiping
Pang et al. (2025)	CP	✓	●			Peg in hole, pouring, drawing
Stranghöner et al. (2025)	CP	✓	●			Connector assembly
Wu et al. (2025)	DG	✓	●			Part transport, picking, long horizon kitchen tasks
Xu et al. (2025)	DG	✓	●			Openbox, steam bun, upright mug
Li et al. (2024a)	CP	✓	●			Pushing, throwing
Liu et al. (2024)	DV	✓			●	Nut assembly, tool hang, gear insertion, coffee pad packing
Wakabayashi et al. (2024)	DV	✓	●			Dish washing
Liu et al. (2023)	DV		●			Driving, atari games
Mandlekar et al. (2023)	DV	✓	●			Tool hang, stacking, insertion, coffee machine
Sun et al. (2023)	CP		●			Driving
Chisari et al. (2022)	DV	✓			●	Close microwave, push button, take of lid, unplug, push, reach, pickup, pull
Parnichkun et al. (2022)	DV	✓	●			Cartpole, navigation
Spencer et al. (2022)	CP				●	Driving
Hoque et al. (2021)	DV	✓	●			Locomotion, fabric manipulation
Kahn et al. (2021)	DV	✓		●		Navigation
Luo et al. (2021)	DV	✓	●			Plug-insertion
Mandlekar et al. (2020)	DV		●			Threading, coffee machine
Pérez-Dattari et al. (2020)	DV	✓	●			Cartpole, driving
Spencer et al. (2020)	CP	✓			●	Driving
Celemin et al. (2019)	CP	✓	●			Ball-in-cup, reaching, writing
Celemin and Ruiz-del-Solar (2019)	CP	✓	●			Cartpole, mountain car, ball dribbling, balancing
Kelly et al. (2019)	CP	✓	●			Driving
Jevtić et al. (2018)	CP	✓	●			Obstacle avoidance
Le et al. (2018)	DV			●		Atari games, maze navigation
Christiano et al. (2017)	DV			●		Atari games, locomotion
Macglashan et al. (2017)	CP	✓		●		Push-pull, ball following, navigation
Ewerton et al. (2016)	CP	✓	●			Reaching
Ross et al. (2011)	CP		●			Atari games
Bradley Knox and Stone (2008)	CP			●		Tetris games

Policy representation (PR), Real-world experiments (RW), Corrective feedback (CF), evaluative feedback (EF).

CP: Classical parametric - linear models, GMM, RBF, DMP/ProMP, shallow MLP.

DV: Deep vision-conditioned - CNN/LSTM that ingest images or point clouds.

DG: Diffusion/generative - score-based or flow models that explicitly model multimodality.

**FIGURE 4:**
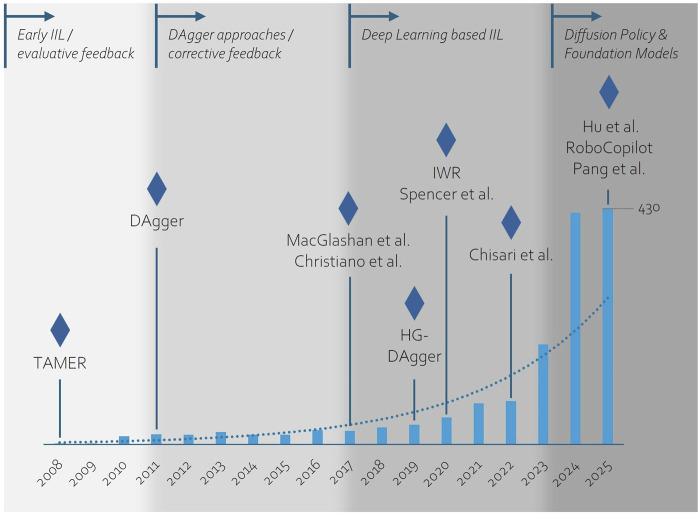
Timeline of IIL research, showing key methodological phases overlaid with the annual number of IIL publications (data from scopus database), highlighting landmark works that helped drive growth in the field.

We summarized the development of IIL approaches visually as a roadmap in Figure 4, highlighting both the growth of research activity and major methodological milestones. The bars indicate the number of IIL-related publications per year based on a search on the Scopus database, showing a steady rise that accelerates in recent years. Overlaid on this trend, we highlighted key phases and representative works that likely contributed to the increasing momentum in this field.

The IIL publications in this section showed that human feedback is integrated in different ways—ranging from purely evaluative signals to corrective cues, and also a hybrid of both. While these approaches have yielded promising results on tasks such as navigation or simple manipulation, transferring them to dexterous manipulation introduces a set of unique challenges, such as much higher action dimensionality, rich and discontinuous contact dynamics, and more challenging demonstration and correction collection. IIL presents a promising avenue for advancing robotic capabilities, particularly in dexterous manipulation. Corrective feedback offers rich, intuitive, and safe guidance but necessitates experienced teachers and relies heavily on their performance. Evaluative feedback, while accessible to non-domain experts and easier to combine with recent trends of large language models, demands more data and interactions, posing potential safety risks. Including reward functions enables self-optimization, yet it comes with the high cost of reward engineering and inherent safety concerns due to autonomous exploration. Balancing these feedback mechanisms is crucial for developing robust and efficient IIL systems adapting to complex real-world scenarios.

## 5 Conclusion

In this survey, we explored the current research landscape of Interactive Imitation Learning (IIL) for dexterous manipulation and identified a notable gap in concrete studies within this domain. We began by discussing the key challenges of dexterous manipulation, including its high-dimensional action space, multi-modal state representations, and long-horizon task complexity. We then presented various approaches for tackling real-world dexterous manipulation, such as imitation learning, reinforcement learning, and IIL, highlighting both their potential and limitations. Given the scarcity of research explicitly addressing IIL for dexterous manipulation, we extended our review to IIL methods used in other robotic applications, explaining how different approaches incorporate human feedback. IIL for dexterous manipulation is an emerging field with substantial opportunities for further research. Notably, the use of on-policy corrections in IIL has proven effective in mitigating covariate shift while enhancing sample efficiency, making it a promising direction for future advancements in dexterous robotic control. As the development of humanoid robotics continues to advance rapidly, the demand for efficient algorithms to equip these robots with the requisite skills is expected to increase significantly in the coming years. The growing interest among industrial companies in utilizing humanoid robots in production and logistics especially underscores the necessity for algorithms that can meet the industry’s specific requirements, such as efficiency, flexibility, and real-world applicability. This trend is further amplified by the recent surge in companies developing dexterous robotic hands, reflecting a broader shift toward enabling fine-grained manipulation capabilities in real-world settings. Consequently, these factors must be accorded a higher priority in developing algorithms than was previously the case in the research field of humanoid robots. In particular, IIL approaches based on diffusion policies and those that use both corrective and evaluative feedback can provide a successful direction here. As task complexity increases, the importance of algorithms capable of handling long-horizon policies grows; thus, the integration of hierarchical approaches becomes crucial.

In summary, while IIL for dexterous manipulation is still in its early stages, it holds great promise for enabling more efficient, scalable, and human-aligned robotic learning. To bring IIL to real-world dexterous tasks, policy representations need to cope with more than 20-DoF hand kinematics, and multimodal encoders with contrastive objectives must fuse visual, tactile, and proprioceptive signals. Beyond these representations, dexterous IIL further requires feedback that operates in structured hand- and contact—state spaces—specifying grasp types, contact modes, and manipulation phases rather than only low—level actions or scalar trajectory preferences—and intuitive interfaces that let humans provide such feedback with minimal cognitive and physical burden. This survey aims to serve as a foundation for future research and innovation in this exciting and rapidly evolving field.

## Footnotes

1
https://www.figure.ai/

2
https://www.1x.tech/

3
https://www.tesla.com/en_eu/AI

4
https://www.scopus.com/

5
https://ieeexplore.ieee.org/

6
https://developer.nvidia.com/isaac/sim

7
https://genesis-embodied-ai.github.io/
